# Racial differences in institutional trust and COVID-19 vaccine hesitancy and refusal

**DOI:** 10.1186/s12889-021-12195-5

**Published:** 2021-11-16

**Authors:** Anisah B. Bagasra, Sara Doan, Christopher T. Allen

**Affiliations:** 1 Department of Psychological Science, Kennesaw State University, 440 Bartow Ave NW, 30144 Kennesaw, GA USA; 2 Department of Technical Communication & Interactive Design, Kennesaw State University, Kennesaw, GA USA

**Keywords:** Trust, Race, Scientific community, Government, Vaccine hesitancy, Vaccine refusal

## Abstract

**Background:**

Previous research has indicated that demographic differences affect COVID-19 vaccination rates. Trust, in both the vaccine itself and institutional trust, is one possible factor. The present study examines racial differences in institutional trust and vaccine status among a nationally representative sample of adults in the United States.

**Methods:**

Data for the current study was collected as part of Wave 8 Omnibus 2000 survey conducted by RAND ALP and consisted of 2080 participants. Responses were collected through the online RAND ALP survey in March 2021.

**Results:**

Trust in the scientific community was the strongest predictor for already receiving at least one dose of the COVID-19 vaccine at the time of study. Asians had a significantly higher trust in the scientific community compared to all other groups. Results also showed a significant difference in level of trust of the government’s response to the COVID-19 pandemic with Indian/Alaskan Natives reporting lower trust compared to Whites, Blacks and Asians. Asians also had a significantly higher level of trust when compared to those who identified as racial Other. Those who identify as American Indian/Alaskan Natives had the lowest levels of institutional trust. Trust in the government’s response was not indicative of vaccination within the sample.

**Conclusions:**

Strategies to increase trust of the scientific community can be employed to address vaccine hesitancy through community-based initiatives and building of partnerships between the scientific community and local community stakeholders.

## Background

The COVID-19 pandemic has claimed the lives of more than 600,000 people in the United States alone and more than 4 million people globally [1, 2]. Despite the availability of three vaccines in the United States and mass vaccination campaigns, the rate of vaccination has stagnated and decreased from peak vaccination rates in February 2021 [3]. Strategies on the state and local levels to increase vaccination rates vary greatly following initial national goals to vaccinate the most vulnerable populations. Current vaccination programs have fully expanded to populations 12 years and older. Factors that potentially play a role in vaccine hesitancy and refusal, such as political affiliation, perceived threat of COVID-19, trust in the vaccine itself, and trust in public institutions involved in vaccine production and distribution [4–7] influence both the ongoing COVID-19 pandemic and responses to future public health crises. Demographic factors, particularly ethnicity and socio-economic status also impact vaccine hesitancy [8].

Furthermore, racial disparities in vaccination rates are of increasing concern, as racial and ethnic minorities have been disproportionately affected by COVID-19 in both severity of infection and mortality rates [9–12]. As of July 2021, approximately 61% of White Americans reported being vaccinated, but only 15% of Hispanics, 9% of Blacks, 6% of Asians and 9% who fall into other racial categories [1]. Racial disparities in vaccine rates are influenced by institutional trust. Institutional trust such as trust in the government and scientific/medical community are significant factors in promoting preventative health behaviors such as compliance with lockdowns and vaccination uptake [13–15]. Low institutional trust was associated with lower likelihood of vaccination in a study conducted in Italy [16]. In the United States, government’s ineffectual early response to the pandemic and inconsistent messaging from the scientific community at the start of the COVID-19 pandemic have been cited by some as justification for their lack of trust in the available COVID-19 vaccines [17].

Medical mistrust encompasses a broad spectrum of mistrust, including the overall health care system, medical research and researchers, health care providers, insurers, and public health officials. This mistrust can lead to under-utilization of health care services, a lack of satisfaction with available services or providers, low adherence to medical advice, and reduced compliance with recommended health behaviors [18, 19]. Racial disparities and other impacts of racism have been linked to rates of medical mistrust [20–22]; as such, higher perceived discrimination correlates with higher medical mistrust [23, 24].

The need to address trust in order to mitigate the continued impact of COVID-19 especially in minority communities already impacted by health disparities has been recognized in recent literature [25, 26]. However, the relationship between institutional mistrust and vaccine hesitancy deserves more attention [27, 28], especially in relation to the COVID-19 pandemic. Trust in public health figures such as Dr. Fauci has been associated with higher likelihood to vaccinate or encourage others to vaccinate [29]. Public health professionals acknowledge that mistrust of both health care services and messaging is a major barrier to effectively engaging minority communities in prevention efforts [30, 31]. Given this limited data, further research is needed to understand the impact of trust on current rates of COVID-19 vaccination.

This study examines racial/ethnic differences in vaccine hesitancy and refusal, ethnic/racial differences in institutional trust, and the impact of institutional trust on vaccination behavior. Institutional trust was divided into two categories: trust in the government’s response to the COVID-19 pandemic and trust in members of the scientific community. Based upon the findings of previous studies, we hypothesized that lower trust in the government would be associated with vaccine hesitancy, while higher trust in the scientific community would predict greater willingness to receive the COVID-19 vaccine. Additionally, based upon previous literature that indicates high rates of medical mistrust in the African American community, we hypothesized that African Americans would report lower trust in the scientific community and higher rates of vaccine hesitancy than other racial/ethnic groups in the sample.

## Methods

Wave 8 of RAND’s American Life Panel (ALP) Omnibus 2000 survey was fielded from March 1st – April 1st, 2021. The ALP is a probability-sampled internet-based panel study designed to represent U.S. adults age 18 and older (for details, please see https://www.rand.org/research/data/alp/panel.html). Informed consent was obtained from all study participants. Data for the current study was collected using items the authors included as part of the Wave 8 Omnibus 2000 survey. Responses to author included items were fielded between March 8th – 19th, 2021 and analyses were conducted in June 2021. All data collected using ALP participants are made available to researchers who register with RAND following a 12-month embargo; data for the current study can be found at: https://alpdata.rand.org/index.php?page=data&p=showsurvey&syid=569. Of the 3391 invited panelists, 2080 (61.3%) responded. The final sample contained 52% women, (M = 51.1 years old; SD = 15.6 years); 75.2% White, 12.2% Black, 7.4% Other, 3.4% Asian or Pacific Islander, 1.9% American Indian or Alaskan Native; 18.2% Latinx; 62.7% with educational attainment of an associate degree or less, 44.2% with a combined family income of $59,999 or less during the previous 12 months, an average household size of 2.82 people (SD = 1.50), and 94.1% covered by some form of health insurance. All study materials and procedures were approved by the Kennesaw State University IRB (OHRP # IRB00001469) and the RAND Human Subjects Protection Committee and the study was conducted in accordance to the Declarations of Helsinki.

### Measures

#### Trust in Information from the scientific community (SCItrust)

Respondents were asked to respond to the following item, “Information from the scientific community (e.g., doctors, nurses, public health professionals) is trustworthy,” on a scale of 1 (*definitely false*) to 5 (*definitely true*); M = 3.71, SD = 1.00).

#### Trust in Government Action Regarding COVID-19 (GOVtrust)

Respondents were asked to respond to the following item, “How much do you believe that the government’s actions concerning the COVID-19 pandemic will be in your personal best interest?” on a scale of 1 (*not at all*) to 4 (*very much*); M = 2.49, SD = 0.97).

#### COVID-19 vaccine hesitancy & refusal

Respondents were asked to respond to the following item, “Which of the following best describes your actions regarding the COVID-19 vaccine?” by choosing from the following options: *I have already received at least one dose of a federally approved COVID-19 vaccine*; *I have scheduled an appointment to get a COVID-19 vaccine*; *I am planning to get a COVID-19 vaccine but do not yet have an appointment*; *I am not planning to get a COVID-19 vaccine*; *I have not yet decided about getting a COVID-19 vaccine*. For regression analyses, data were recoded to create two dichotomous variables reflecting *vaccine hesitancy* (i.e., “*I have not yet decided about getting a COVID-19 vaccine”*) and vaccine refusal. (i.e., “*I am not planning to get a COVID-19 vaccine”*) coded as 0 = false and 1 = true.

### Data analysis

An ANOVA of COVID-19 vaccination status by race was conducted to identify statistically significant differences with Tukey’s HSD post-hoc tests. An independent-samples t-test of COVID-19 vaccination status by Latinx identity was conducted to identify statistically significant differences.

Separate logistic regression models were estimated using Bayesian estimation in Mplus version 8.6 software program [32] and following current best practices in Bayesian inference [33] to examine the concurrent effects of demographics and trust on vaccination hesitancy and refusal, respectively. The following demographic variables were included as covariates in the model: U.S. Census region, Rural (yes/no), gender, age, White (yes/no), Latinx (yes/no), education, family income, household size. SCItrust and GOVtrust were included in the model as the main predictors of interest. COVID-19 Vaccination Status was included in the model as the outcome variable. Regression model fit will be assessed holistically using both the posterior predictive *P*-value (PPp). PPp range from 0 to 1, with a value of .50 considered perfect model fit. PPp Values of less than .10, or greater than .90, suggest a poor model fit with data [34].

## Results

### Descriptives – institutional trust by race & ethnicity

Mean scores for trust of the scientific community and government’s response to COVID-19 by race and ethnicity are shown below in Table 1. Asians had a significantly higher trust in the scientific community compared to all other groups. There was also a significant difference in trust of the government’s response to the COVID-19 pandemic when comparing American Indian/Alaskan Natives to Whites, Blacks and Asians. Asians also had a significantly higher level of trust when compared to those who identified as racial Other. Those who identify as American Indian/Alaskan Natives had the lowest levels of institutional trust.

**Table 1:** Racial differences in institutional trust

Institution	Race				
	White	Black	Native	Asian	Other
Scientific Community	3.73^a^	3.56^b^	3.47^a,b^	4.04^c^	3.69^a,b^
Government	2.48^a,c^	2.57^a,c^	2.10^b,c^	2.68^a^	2.40^a,c^

*Note: Values with non-matching superscripts are statistically different at p < .05*

### Descriptives – COVID-19 vaccine status by race & ethnicity

Percentages for each vaccination status category by race and ethnicity are shown below in Table 2. American Indian or Alaskan Native participants had the highest rates of vaccine refusal (i.e., “Not Planning to Get Vaccinated”). Other race participants had the highest rates of vaccine hesitancy (i.e., “Undecided About Getting Vaccinated”).

**Table 2:** COVID-19 vaccination status by race & ethnicity

Race	Vaccinated	Has Vaccine Appointment	Plans to Get Vaccinated	Not Planning to Get Vaccinated	Undecided About Getting Vaccinated
White	35.8%	5.1%	26.9%	19.5%	12.6%
Black	28%	4.8%	24.1%	15.3%	27.9%
Other	22.6%	1.6%	24.7%	22.9%	28.3%
Asian or Pacific Islander	0%	0%	60.5%	16.5%	8.8%
American Indian or Alaskan Native	16.1%	0%	8.0%	60.6%	15.4%
Latinx	26.2%	7.2%	18.4	27.4%	20.7%

### COVID-19 vaccine status by race

Results of the ANOVA showed that the effect of race was significant F (4, 2073) = 14.07, *p* < .001. Post hoc analyses using the Tukey HSD post hoc criterion for significance indicated that White participants reported significant differences in their COVID-19 vaccine status (M = 2.68, SD = 1.44) than Black (M = 3.10, SD = 1.56), American Indian or Alaskan Native (M = 3.59, SD = 1.25), and Other race (M = 3.33, SD = 1.48) participants but, not Asian (M = 3.06, SD = 1.05) participants; η^2^ = .026, 95% CI [.013, 040]. There were no statistically significant differences in COVID-19 vaccine status among non-White participants.

### COVID-19 vaccine status by Latinx identity

Results of the t-test showed a significant effect of Latinx identity, t (549.055) = − 4.118, *p* < .001, with Latinx identified (M = 3.09, SD = 1.49) participants being less likely to be vaccinated than non-Latinx (M = 2.75, SD = 1.45) participants; *d* = −.24, 95% CI [−.349, −.126].

### Regression model – vaccine hesitancy

The model that regressed vaccine hesitancy on demographics, SCItrust, and GOVtrust demonstrated excellent model fit: PPp = .52, 95% Credibility Interval [− 11.72, 21.22].

After controlling for demographics, the Bayesian logistic regression model indicated that U.S. adults’ trust in information from the scientific community is a stronger predictor (β = −.16; see Table 3) than trust in government actions (β = −.11) of COVID-19 vaccine hesitancy at the time of data collection (March 2021). Specifically, for both SCItrust and GOVtrust, less trust was associated with a greater likelihood of vaccine hesitancy.

**Table 3:** Bayesian regression model results (standardized): vaccine hesitancy

Predictor	Estimate	Posterior *SD*	*p* *(one-tailed)*	95% CI	
				*LL*	*UL*
**Gender** (male is reference group)	0.078	0.033	0.018^*^	0.004	0.143
**Age**	−0.195	0.039	0.000^*^	− 0.274	− 0.121
**Education**	− 0.104	0.039	0.002^*^	−0.191	− 0.035
**White** (not White is reference group)	−0.078	0.030	0.002^*^	−0.141	−0.024
Latinx (not Latinx is reference group)	0.000	0.032	0.504	−0.064	0.062
Household Size	−0.012	0.031	0.353	−0.074	0.045
**Total Family Income**	−0.147	0.035	0.002^*^	−0.211	−e0.078
**Trust in Information from the Scientific Community**	−0.155	0.036	0.000^*^	−0.230	−0.088
**Trust in Government Actions Regarding COVID-19**	−0.107	0.039	0.002^*^	−0.184	−0.032
**Model** *R*^2^	0.211	0.025	0.000^*^	0.1563	0.258

*Note*: *CI* credible interval, *LL* lower limit, *UL* upper limit; ^*^ = significant at *p* < .025

### Regression model – vaccine refusal

The model that regressed vaccine refusal on demographics, SCItrust, and GOVtrust demonstrated excellent model fit: PPp = .51, 95% Credibility Interval [−10.69, 19.74].

After controlling for demographics, the Bayesian logistic regression model indicated that U.S. adults’ trust in information from the scientific community is a stronger predictor (β = −.33; see Table 4) than trust in government actions (β = −.30) of COVID-19 vaccine refusal at the time of data collection (March 2021). Specifically, for both SCItrust and GOVtrust, less trust was associated with a greater likelihood of vaccine refusal.

**Table 4:** Bayesian regression model results (standardized): vaccine refusal

Predictor	Estimate	Posterior *SD*	*p* *(one-tailed)*	95% CI	
				*LL*	*UL*
Gender (male is reference group)	0.004	0.031	0.437	−0.059	0.063
**Age**	−0.225	0.033	0.000^*^	−0.296	−0.164
**Education**	−0.099	0.034	0.000^*^	−0.171	−0.035
White (not White is reference group)	−0.005	0.030	0.441	−0.066	−0.052
Latinx (not Latinx is reference group)	0.011	0.029	0.349	−0.050	0.065
Household Size	−0.001	0.031	0.485	−0.064	0.060
Total Family Income	−0.018	0.033	0.289	−0.085	−0.042
**Trust in Information from the Scientific Community**	−0.333	0.030	0.000^*^	−0.399	−0.278
**Trust in Government Actions Regarding COVID-19**	−0.304	0.032	0.000^*^	−0.362	−0.239
**Model** *R*^2^	0.452	0.027	0.000^*^	−2.864	−1.976

*Note*: *CI* credible interval, *LL* lower limit, *UL* upper limit; ^*^ = significant at *p* < .025

## Discussion

Findings from the present study suggest that there are racial differences in vaccine hesitancy and vaccine refusal, and significant ethnic differences (Latinx vs non-Latinx identity) in vaccination rates at the time of this study. Additionally, there are racial differences in trust of the scientific community where lower trust in the scientific community was a stronger indication of higher vaccine hesitancy and refusal in the study sample. Trust in the government’s response was not indicative of vaccination status within the sample, differing from some previous research on government trust and COVID-19 behavioral compliance in other countries [16] and studies of government trust and vaccine intention in previous pandemics [15]. Further research needs to be conducted to understand why members of the African American community, Latinx communities, and those identifying as Other express higher vaccine hesitancy than Whites and Asians. The study also identified American Indian/Alaska Native members of the sample as having the highest vaccine refusal rate compared to other groups, as well as the lowest level of institutional trust. In the study, we did not differentiate between tribal governments, which have high vaccination rates [35], and U.S. state or federal governments. Little research has focused on this community and their concerns throughout the pandemic, with most efforts focused on creating health infrastructure on reservations to mitigate the spread of COVID-19 [36].

Approaches to addressing vaccine hesitancy may need to be markedly different within different communities. Razai and colleagues [37] identify the five Cs that impact vaccine uptake: confidence in vaccine safety and efficacy, complacency regarding COVID-19 infection risks, convenience of access, communication, and context. Listening to communities to determine the root causes is an important strategy moving forward. Conducting community-based research, such as focus groups embedded within specific communities, can tease out more specific reasons given for vaccine hesitancy. Given ethnic differences in vaccine rates, all five of the “Cs” need to be analyzed to determine if there are greater structural barriers to vaccination such as time off from work, easy access to vaccination sites, and language barriers, or issues with vaccine confidence and complacency. Vaccine hesitancy may be addressed by increasing confidence in the vaccine, discussing concerns about side effects, and highlighting vaccine efficacy in preventing COVID-19 and reducing the severity of COVID-19 infection [38, 39].

Trust of the scientific community was the strongest predictor for already receiving at least one dose of the COVID-19 vaccine at the time of study, suggesting that strategies to increase trust of the scientific community can be employed to address vaccine hesitancy. Results also suggest that members of the scientific community including doctors, researchers, and other health care professionals may be viewed as credible messengers by community members. Opel, Lo, and Peek [40] have outlined vaccine communication strategies to address mistrust during clinical encounters. These strategies address historical injustices and personal feelings of distrust. Such strategies are vital, especially in communities that have historically been excluded from medical care and scientific research such as American Indians and African Americans. Historical incidents of oppression cannot be ignored in efforts to build trust between community partners and medical providers. Increasing health literacy and understanding of the scientific process through which vaccine development takes place is another important element of increasing trust.

Given the rapid spread of misinformation regarding COVID-19 on social media platforms, social media can also be an effective vehicle to address different aspects of mistrust. Debunking misinformation and increasing media literacy and health literacy can also contribute to improved trust [41, 42]. Drawing on scientists’ and medical professionals’ expertise when creating short video clips in language for non-experts can combat misinformation and make the vaccine development process more transparent for the hesitant. For example, Louisiana State University tweeted a three-minute video, titled “Dr. Catherine O’Neal Sets the Record Straight,” discussing the rise of COVID variants, using a football training metaphor to explain how scientists developed MRNA vaccines quickly via available technology; the video has over 3500 likes on Twitter [43]. Explaining the changing recommendations regarding COVID-19 protective behaviors throughout the pandemic can also counter the current lack of trust impacting vaccine acceptance. Discussions within communities regarding the scientific process can increase understanding of why messaging changes as new information became available.

All approaches to address trust should be community-based and involve partnerships between community stakeholders and trusted members of the medical community. Community-based initiatives that engage local members of medical communities with other community leaders to start dialogues on past causes of medical mistrust in minority communities, especially within Native American and African American communities, is necessary. Community leaders who are viewed as trusted and credible messengers within their communities should have equal partnerships with public health agencies in decision making regarding vaccine distribution sites and campaigns, with plenty of time leading up to the vaccine drive to address concerns among the vaccine hesitant.

### Limitations

The present study did not examine specific factors that may have impacted vaccine hesitancy such as fear of side effects of the vaccine and logistical challenges. Additionally, the study was conducted in March 2021, only a few months into vaccine availability, and before vaccination opened to all adults sixteen and older regardless of age and health risk status. These factors may impact vaccine status and acceptance of the vaccine. As of this writing, data suggests that 99% of COVID-19 deaths in the United States are among unvaccinated individuals, bolstering further evidence of vaccines’ efficacy to prevent COVID-19 related mortality [44].

Medical mistrust continues to be a significant barrier to adoption of recommended health prevention behaviors, as evidenced in previous studies and the current findings applied to the COVID-19 vaccine. Increasing trust in the medical community can contribute to their advice being viewed as credible and more likely to result in behavioral engagement. Findings from this study demonstrate that rates of vaccination vary among different racial groups, and trust in the scientific community is a predictor of vaccination. Medical mistrust and racial/ethnic differences in trust are important factors that must be considered in public health strategies to increase vaccination rates.

## Data Availability

All data collected using ALP participants are made available (following a 12-month embargo) to researchers who register with RAND; data for the current study can be found at https://alpdata.rand.org/index.php?page=data&p=showsurvey&syid=569

## References

[1] Centers for Disease Control and Prevention (CDC) (2021). Percent of people receiving COVID-19 vaccine by Race/ethnicity and data reported to CDC.

[2] WHO COVID-19 Dashboard. Geneva: World Health Organization; 2020. Available online: https://covid19.who.int/.

[3] Diesel J, Sterrett N, Dasgupta S, Kriss JL, Barry V, Vanden Esschert K (2021). COVID-19 vaccination coverage among adults — United States, December 14, 2020–May 22, 2021. MMWR Morb Mortal Wkly Rep.

[4] Fridman A, Gershon R, Gneezy A. COVID-19 and vaccine hesitancy: A longitudinal study. Capraro V, editor. PLoS One. 2021;16(4):e0250123. 10.1371/journal.pone.0250123 PMC8051771 33861765

[5] Attwell K, Lake J, Sneddon J, Gerrans P, Blyth C, Lee J. Converting the maybes: Crucial for a successful COVID-19 vaccination strategy. Samy AM, editor. PLoS One 2021; 16(1):e0245907. 10.1371/journal.pone.0245907 PMC7817004 33471821

[6] Viswanath K, Bekalu M, Dhawan D, Pinnamaneni R, Lang J, McLoud R (2021). Individual and social determinants of COVID-19 vaccine uptake. BMC Public Health.

[7] Elhadi M, Alsoufi A, Alhadi A, Hmeida A, Alshareea E, Dokali M, Abodabos S, Alsadiq O, Abdelkabir M, Ashini A, Shaban A, Mohammed S, Alghudban N, Bureziza E, Najah Q, Abdulrahman K, Mshareb N, Derwish K, Shnfier N, Burkan R, al-Azomi M, Hamdan A, Algathafi K, Abdulwahed E, Alheerish K, Lindi N, Anaiba M, Elbarouni A, Alsharif M, Alhaddad K, Alwhishi E, Aboughuffah M, Aljadidi W, Jaafari A, Khaled A, Zaid A, Msherghi A (2021). Knowledge, attitude, and acceptance of healthcare workers and the public regarding the COVID-19 vaccine: a cross-sectional study. BMC Public Health.

[8] While A (2021). Evidence-based strategies to promote vaccine acceptance. Br J Community Nurs.

[9] Do DP, Frank R (2021). Unequal burdens: assessing the determinants of elevated COVID-19 case and death rates in new York City’s racial/ethnic minority neighbourhoods. J Epidemiol Community Health.

[10] Dawes D. Health Inequities: A Look at the Political Determinants of Health During the COVID-19 Pandemic. Am J Health Stud. 2020;35(2):77–82. Available from: https://amjhealthstudies.com/index.php/ajhs/article/view/191.

[11] Mackey K, Ayers CK, Kondo KK, Saha S, Advani SM, Young S, Spencer H, Rusek M, Anderson J, Veazie S, Smith M, Kansagara D (2021). Racial and ethnic disparities in COVID-19–related infections, hospitalizations, and deaths: a systematic review. Ann Intern Med.

[12] Coughlin SS, Moore JX, George V, Johnson JA, Hobbs J (2020). COVID-19 among African Americans: from preliminary epidemiological surveillance data to public Health action. Am J Public Health.

[13] Caplanova A, Sivak R, Szakadatova E (2021). Institutional trust and compliance with measures to fight COVID-19. Int Adv Econ Res.

[14] Latkin CA, Dayton L, Strickland JC, Colon B, Rimal R, Boodram B (2020). An assessment of the rapid decline of trust in US sources of public information about COVID-19. J Health Commun.

[15] Sibley CG, Greaves LM, Satherley N, Wilson MS, Overall NC, Lee CHJ, Milojev P, Bulbulia J, Osborne D, Milfont TL, Houkamau CA, Duck IM, Vickers-Jones R, Barlow FK (2020). Effects of the COVID-19 pandemic and nationwide lockdown on trust, attitudes toward government, and well-being. Am Psychol.

[16] Prati G (2020). Intention to receive vaccine against SARS-CoV-2 in Italy and its association with trust, worry, and beliefs about the origin of the virus. Health Educ Res.

[17] Kerr J, Panagopoulos C, van der Linden S (2021). Political polarization on COVID-19 pandemic response in the United States. Personal Individ Differ.

[18] Powell BJ, Fernandez ME, Williams NJ, Aarons GA, Beidas RS, Lewis CC, McHugh SM, Weiner BJ (2019). Enhancing the impact of implementation strategies in healthcare: a research agenda. Front Public Health.

[19] Randolph HE, Barreiro LB (2020). Herd immunity: understanding COVID-19. Immunity.

[20] Jaiswal J, Halkitis PN (2019). Towards a more inclusive and dynamic understanding of medical mistrust informed by science. Behav Med.

[21] Sutton AL, He J, Edmonds MC, Sheppard VB (2019). Medical mistrust in black breast cancer patients: acknowledging the roles of the trustor and the trustee. J Cancer Educ.

[22] Williamson LD, Smith MA, Bigman CA (2019). Does discrimination breed mistrust? Examining the role of mediated and non-mediated discrimination experiences in medical mistrust. J Health Commun.

[23] Bazargan M, Cobb S, Assari S, Kibe LW (2021). Awareness of palliative care, hospice care, and advance directives in a racially and ethnically diverse sample of California adults. Am J Hosp Palliat Med.

[24] Hammond WP (2010). Psychosocial correlates of medical mistrust among African American men. Am J Community Psychol.

[25] Peek ME, Simons RA, Parker WF, Ansell DA, Rogers SO, Edmonds BT (2021). COVID-19 among African Americans: an action plan for mitigating disparities. Am J Public Health.

[26] Davis DJ, Chaney C, BeLue R (2020). Why “we Can’t breathe” during COVID-19. J Comp Fam Stud.

[27] Breakwell GM (2020). Mistrust, uncertainty and health risks. Contemp Soc Sci.

[28] Carney CJJ (2021). The role of experimentation and medical mistrust in COVID-19 vaccine skepticism. Divers Issues High Educ.

[29] Latkin CA, Dayton L, Yi G, Konstantopoulos A, Boodram B (2021). Trust in a COVID-19 vaccine in the U.S.: A social-ecological perspective. Soc Sci Med.

[30] Ferdinand KC (2021). Overcoming barriers to COVID-19 vaccination in African Americans: the need for cultural humility. Am J Public Health.

[31] Jackson DN, Peterson EB, Blake KD, Coa K, Chou W-YS (2019). Americans’ Trust in Health Information Sources: trends and sociodemographic predictors. Am J Health Promot.

[32] Muthén LK, Muthén B (2017). Mplus User’s Guide.

[33] Depaoli S, van de Schoot R (2017). Improving transparency and replication in Bayesian statistics: the WAMBS-checklist. Psychol Methods.

[34] Muthén B, Asparouhov T (2012). Bayesian structural equation modeling: a more flexible representation of substantive theory. Psychol Methods.

[35] Ndugga N, Pham O, Hill L, Artiga S, Alam R, Parker D. Kaiser Family Foundation; 2021. Noah latest data on COVID-19 vaccinations race/ethnicity. https://www.kff.org/coronavirus-covid-19/issue-brief/latest-data-on-covid-19-vaccinations-race-ethnicity/.

[36] Shaw G (2020). Neurologists on the front lines: the burden of COVID-19 on native American communities. Neurol Today.

[37] Razai MS, Oakeshott P, Esmail A, Wiysonge CS, Viswanath K, Mills MC (2021). COVID-19 vaccine hesitancy: the five Cs to tackle behavioural and sociodemographic factors. J R Soc Med.

[38] Chen T, Dai M, Xia S, Zhou Y (2021). Do messages matter? Investigating the combined effects of framing, outcome uncertainty, and number format on COVID-19 vaccination attitudes and intention. Health Commun.

[39] Chu H, Liu S. Integrating health behavior theories to predict American’s intention to receive a COVID-19 vaccine. Patient Educ Couns. 2021;104(8):1878–86. 10.1016/j.pec.2021.02.031. 10.1016/j.pec.2021.02.031 PMC7889032 33632632

[40] Opel DJ, Lo B, Peek ME (2021). Addressing mistrust about COVID-19 vaccines among patients of color. Ann Intern Med.

[41] Doan S (2021). Misrepresenting COVID-19: lying with charts during the second Golden age of data design. J Bus Tech Commun.

[42] Sleigh J, Amann J, Schneider M, Vayena E. Qualitative analysis of visual risk communication on twitter during the Covid-19 pandemic. BMC public health. 2021;21(1):810. 10.1186/s12889-021-10851-4. 10.1186/s12889-021-10851-4 PMC8079223 33906626

[43] @LSU L. Dr. Catherine O’Neal Sets the Record Straight [Tweet]. 2021. https://twitter.com/lsu/status/1423376894636998661.

[44] Johnson CK, Stobbe M. Nearly all COVID deaths in US are now among unvaccinated. AP News. 2021; Available from: https://apnews.com/article/coronavirus-pandemic-health-941fcf43d9731c76c16e7354f5d5e187.

